# Supplementary material for: Shoelaces: an interactive tool for ribosome profiling processing and visualization
Source: BMC Genomics. 2018 Jul 18;19:543. doi: 10.1186/s12864-018-4912-6 (PMC6052522; doi:10.1186/s12864-018-4912-6)
Supplement: Supplementary file 1 — Analysis examples. Figures S1-S3. Three different examples of offset selection (PDF file) for human ribosome profiling datasets: SRR493747 [15], treated with harringtonine and cyclohexamide; SRR1039861 [22], treated with cyclohexamide; SRR592961 [20], no drug. Table S1: Comparison of selected footprint lengths as originally in human ribosome profiling studies and Shoelaces. Figure S4: Comparison of reads mapping to different parts of transcript as selected by Shoelaces and the original manual selection (SRR493747 [15]). (PDF 8213 kb) [file 12864_2018_4912_MOESM1_ESM.pdf]

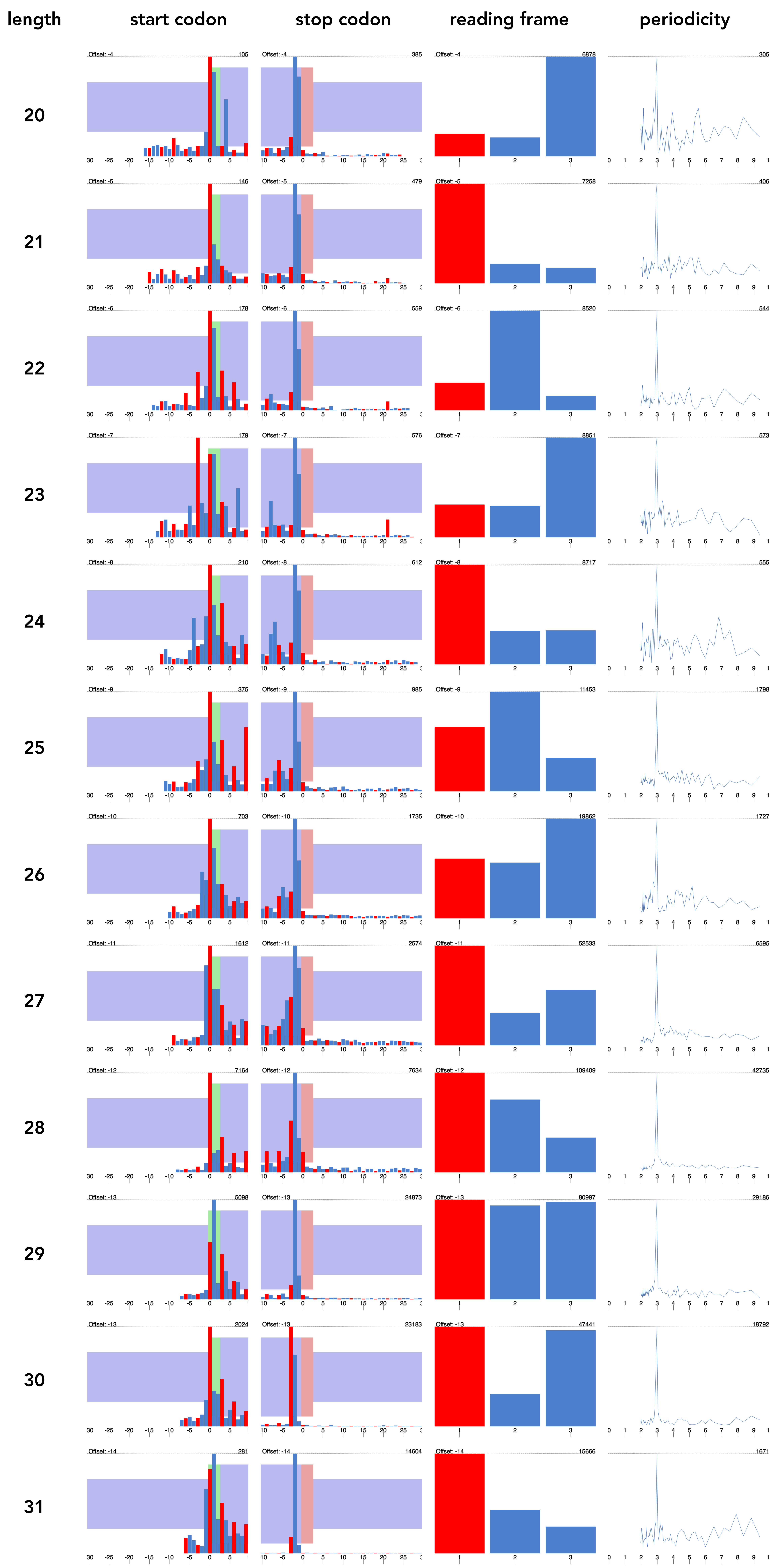

Additional figure 1

Analysis of human ribosome profiling, SRR493747 (Ingolia *et al.*, 2012). The libraries were treated with harringtonine, which causes accumulation of ribosomes at translational initiation sites and cycloheximide, which inhibits translation. The fragments of lengths 20-31 exhibit periodicity. The peaks are clear on start and before stop codons and the offsets change in increments of one nucleotide with increasing footprint length.

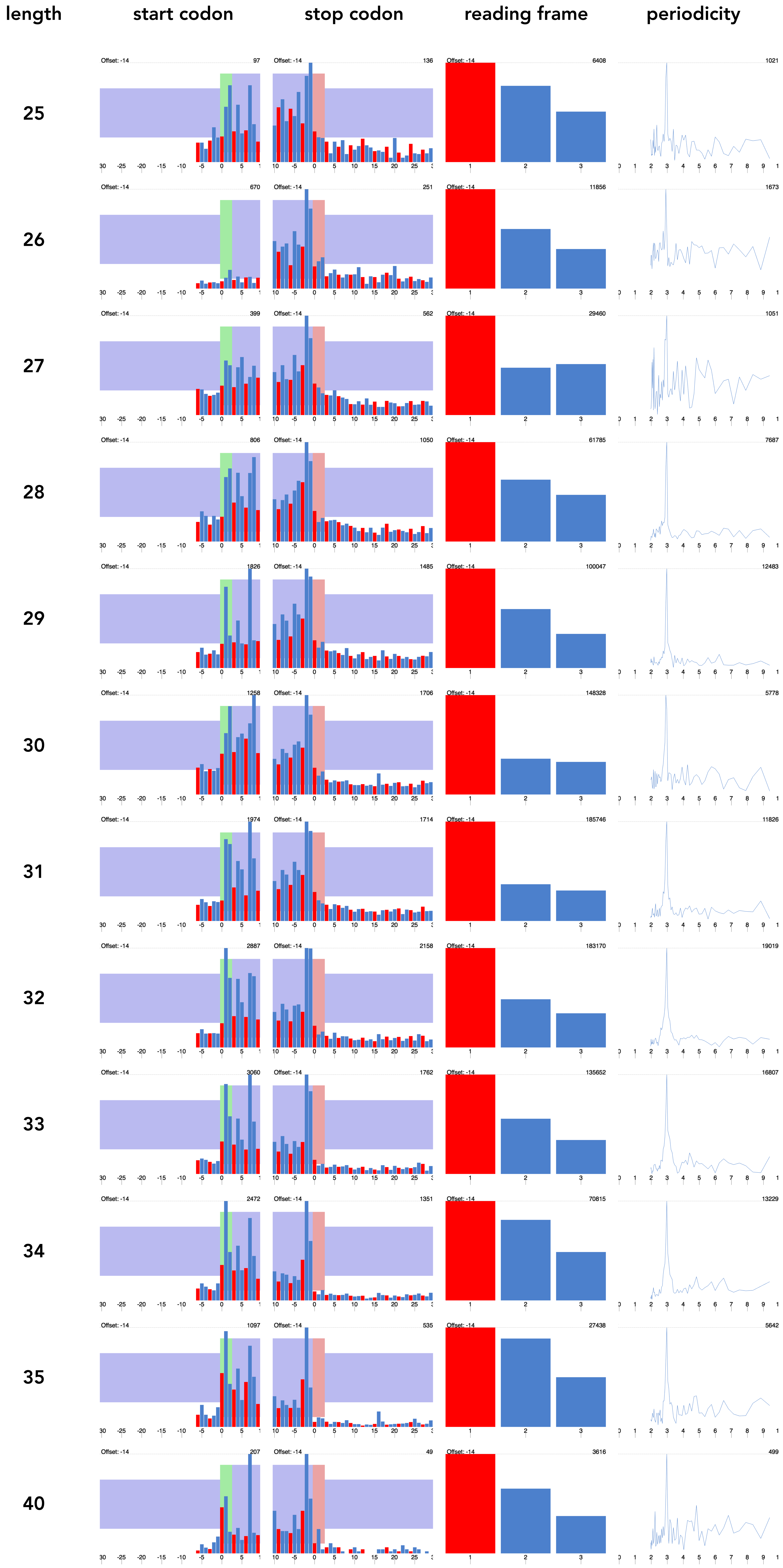

Additional figure 2

Analysis of human ribosome profiling, SRR1039861 (Subtelny *et al.*, 2014). The libraries were treated with cycloheximide, which inhibits translation. The footprints of lengths 25-35 and 40 are periodic (there are no footprints of lengths 36-39 in the library). The peaks are somewhat ambiguous on start, but clear before stop codons and all lengths map preferentially to the first reading frame. The offset from 5′ end of footprint is uniform for all lengths.

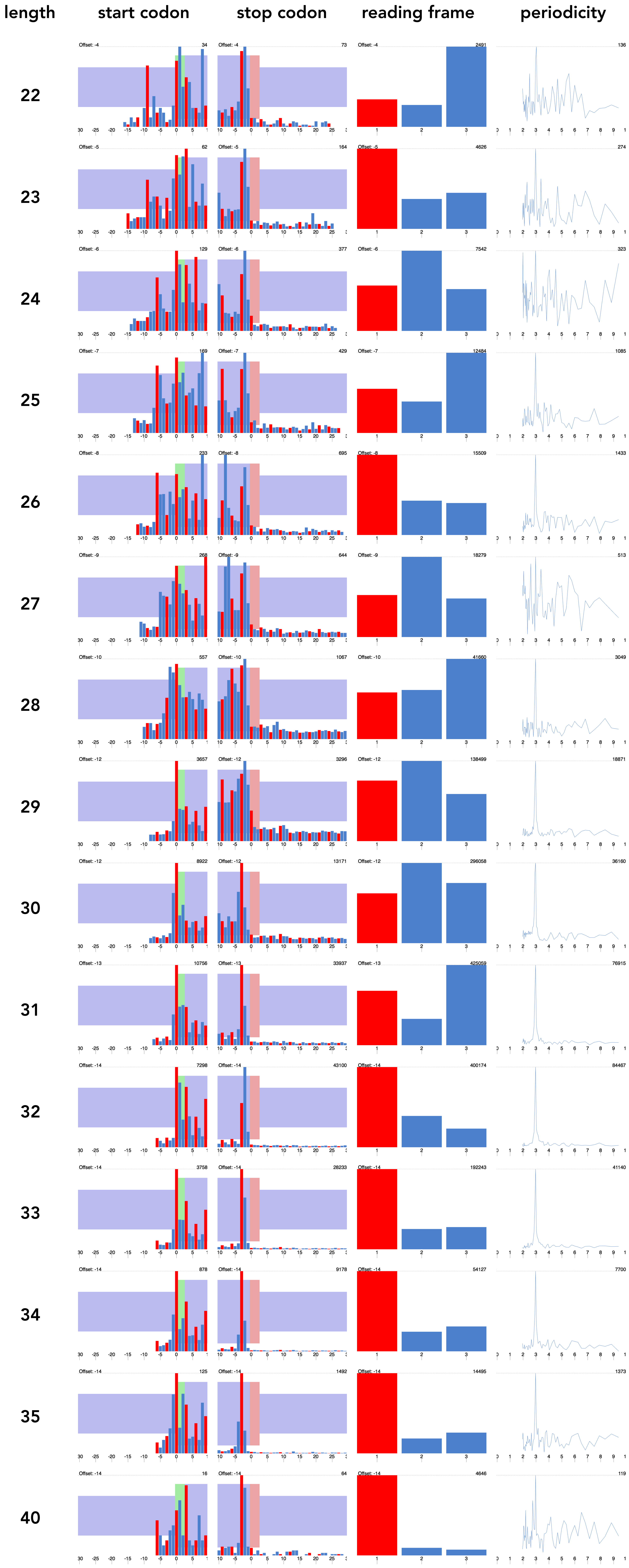

Additional figure 3

Analysis of human ribosome profiling, SRR592961 (Stern-Ginossar *et al.*, 2012). There were no translational inhibitors used in the library preparation. The footprints of lengths 22-35 and 40 are periodic (there are no footprints of lengths 36-39 in the library). The metagene profiles over start codon are unclear for shorter fragment lengths, but clear for the last codon of the coding region. The offsets change in increments of one nucleotide for shorter fragment lengths, while are more uniform for longer footprints. Longer footprints also map preferentially to the first reading frame.

Additional table 1

Comparison of selected footprint lengths as in original study (blue) and based on periodicity in Shoelaces (**S**). Shoelaces captures up to 32% more reads mapping to CDSs and 5'leaders compared to arbitrary manual selection.

Study	Study ID	Run ID	Footprint length																					% gain in reads over CDSs and 5'leaders
			20	21	22	23	24	25	26	27	28	29	30	31	32	33	34	35	36	37	38	39	40	
**Andreev *et al.*, 2015**	SRP038695	SRR1173905	n/a	n/a	n/a	n/a	n/a	n/a	n/a	n/a	S	n/a	n/a	n/a	n/a	S	n/a	n/a	n/a	n/a	n/a	n/a	n/a	**0.1**
		SRR1173907	n/a	n/a	n/a	n/a	n/a	n/a	n/a	n/a	S	n/a	n/a	n/a	n/a	S	n/a	n/a	n/a	n/a	n/a	n/a	n/a	**0.1**
		SRR1173909								S	S	S	S	S	S	S	S	S	S			n/a	n/a	**9.6**
		SRR1173910								S	S	S	S	S	S	S	S	S	S		n/a	n/a	n/a	**9.1**
		SRR1173913								S	S	S	S	S	S	S	S				n/a	n/a	n/a	**15.9**
		SRR1173914	n/a	n/a						S	S	S	S	S	S	S	S	n/a	n/a	n/a	n/a	n/a	n/a	**15.5**
**Gonzalez *et al.*, 2014**	SRP031501	SRR1562539	S	S	S	S	S	S	S	S	S	S	S	S	S	S	S					n/a	n/a	**13.4**
		SRR1562540	S	S	S	S	S	S	S	S	S	S	S	S	S					n/a	n/a	n/a	n/a	**4.1**
		SRR1562541	S	S	S	S	S	S	S	S	S	S	S	S	S				n/a	n/a	n/a	n/a	n/a	**5.4**
		SRR1562542	S	S	S	S	S	S	S	S	S	S	S	S	S		n/a	n/a	n/a	n/a	n/a	n/a	n/a	**6.5**
		SRR1562543		S	S	S	S	S	S	S	S	S	S	S	S			n/a	n/a	n/a	n/a	n/a	n/a	**4.5**
**Guo *et al.*, 2010**	SRP002605	SRR057511						S	S	S	S	S	S	S	n/a	n/a	n/a	n/a	S	n/a	n/a	n/a	n/a	**19.5**
		SRR057512						S	S	S	S	S	S	S	n/a	n/a	n/a	n/a	S	n/a	n/a	n/a	n/a	**19.9**
		SRR057516						S	S	S	S	S	S	S	n/a	n/a	n/a	n/a	S	n/a	n/a	n/a	n/a	**19.2**
		SRR057517						S	S	S	S	S	S	S	n/a	n/a	n/a	n/a	S	n/a	n/a	n/a	n/a	**19.2**
		SRR057521						S	S	S	S	S	S	S	n/a	n/a	n/a	n/a	S	n/a	n/a	n/a	n/a	**16.7**
		SRR057522						S	S	S	S	S	S	S	n/a	n/a	n/a	n/a	S	n/a	n/a	n/a	n/a	**16.7**
		SRR057526						S	S	S	S	S	S	S	n/a	n/a	n/a	n/a	S	n/a	n/a	n/a	n/a	**22.1**
		SRR057529						S	S	S	S	S	S	S	n/a	n/a	n/a	n/a	S	n/a	n/a	n/a	n/a	**23.0**
		SRR057532						S	S	S	S	S	S	S	n/a	n/a	n/a	n/a	S	n/a	n/a	n/a	n/a	**23.0**
		SRR065774	n/a	n/a	n/a			S	S	S	S	S	S	S	n/a	n/a	n/a	n/a	S	n/a	n/a	n/a	n/a	**8.2**
		SRR065775	n/a					S	S	S	S	S	S	S	n/a	n/a	n/a	n/a	S	n/a	n/a	n/a	n/a	**8.0**
		SRR065779	n/a	n/a	n/a			S	S	S	S	S	S	S	n/a	n/a	n/a	n/a	S	n/a	n/a	n/a	n/a	**10.1**
		SRR065780	n/a					S	S	S	S	S	S	S	n/a	n/a	n/a	n/a	S	n/a	n/a	n/a	n/a	**10.0**
**Hsieh *et al.*, 2012**	SRP010679	SRR403883	S	S	S	S	S	S	S	S	S	S	S	S					n/a	n/a	n/a	n/a		**1.9**
		SRR403885	S	S	S	S	S	S	S	S	S	S	S	S					n/a	n/a	n/a	n/a	n/a	**3.1**
		SRR403887	S	S	S	S	S	S	S	S	S	S	S	S	S	S	S	S	n/a	n/a	n/a	n/a	S	**5.1**
		SRR403889	S	S	S	S	S	S	S	S	S	S	S	S	S	S	S	S	n/a	n/a	n/a	n/a		**1.1**
		SRR403891	S	S	S	S	S	S	S	S	S	S	S	S	S				n/a	n/a	n/a	n/a		**1.6**
		SRR403893			S	S	S	S	S	S	S	S	S	S	S	S	S	S	n/a	n/a	n/a	n/a	S	**0.9**
**Ingolia *et al.*, 2012**	SRP012648	SRR493747	S	S	S	S	S	S	S	S	S	S	S	S								n/a	n/a	**7.2**
		SRR493748	S	S	S	S	S	S	S	S	S	S	S	S	S							n/a	n/a	**6.5**
		SRR493749	S	S	S	S	S	S	S	S	S	S	S	S	S							n/a	n/a	**8.1**
**Ingolia *et al.*, 2014**	SRP045257	SRR1536302		S	S	S	S	S	S	S	S	S	S			S	S	S	S	S	n/a	n/a	n/a	**30.9**
		SRR1536303		S	S	S	S	S	S	S	S	S	S			S	S	S		n/a	n/a	n/a	n/a	**25.9**
		SRR1536304	S	S	S	S	S	S	S	S	S	S	S	S	S	S	S	S			n/a	n/a	n/a	**7.3**
		SRR1536305	S	S		S	S	S	S	S	S	S	S	S	S	S			n/a	n/a	n/a	n/a	n/a	**6.6**
**Lee *et al.*, 2012**	SRP014629	SRR618770			S	S	S	S	S	S	S	S	S	S	S	S		n/a	n/a	n/a	n/a	n/a	n/a	**32.4**
		SRR618771	S	S	S	S	S	S	S	S	S	S	S	S	S	S	S		n/a	n/a	n/a	n/a	n/a	**28.6**
		SRR618772	n/a	n/a	n/a	S	S	S	S	S	S	S	S	S				n/a	n/a	n/a	n/a	n/a	n/a	**28.0**
		SRR618773	n/a	S	S	S	S	S	S	S	S	S	S	S				n/a	n/a	n/a	n/a	n/a	n/a	**24.7**
		SRR964946	n/a	n/a			S	S	S	S	S	S	S	S				n/a	n/a	n/a	n/a	n/a	n/a	**17.3**
**Liu *et al.*, 2013**	SRP017263	SRR619082	S	S	S	S	S	S	S	S	S	S	S	S	S			n/a	n/a	n/a	n/a	n/a	n/a	**5.6**
		SRR619083		S	S	S	S	S	S	S	S	S	S	S	S	S	S		n/a	n/a	n/a	n/a	n/a	**2.0**
		SRR619084	S	S	S	S	S	S	S	S	S	S	S	S	S	S	S	n/a	n/a	n/a	n/a	n/a	n/a	**5.6**
		SRR619085	S	S	S	S	S	S	S	S	S	S	S	S	S	S	S	S	n/a	n/a	n/a	n/a	n/a	**1.1**
		SRR619086	S	S	S	S	S	S	S	S	S	S	S	S	S	S	n/a	n/a	n/a	n/a	n/a	n/a	n/a	**4.1**
		SRR619087				S	S	S	S	S	S	S	S	S	S		n/a	n/a	n/a	n/a	n/a	n/a	n/a	**3.0**
		SRR619088	n/a	n/a		S	S	S	S	S	S	S	S	S	S	S		S	S		S		n/a	**1.0**
		SRR619089				S	S	S	S	S	S	S	S	S	S	S		S	S		S			**0.9**
		SRR619090					S	S	S	S	S	S	S	S	S	S		S	S	S	S	S		**0.4**
		SRR619091					S	S	S	S	S	S	S	S	S	S		S	S	S	S	S		**0.4**
		SRR619092	S			S	S	S	S	S	S	S	S	S	S				S			S		**-2.8**
		SRR619093				S	S	S	S	S	S	S	S	S	S			S	S	S	S	S		**-1.4**
		SRR619094	S	S		S	S	S	S	S	S	S	S	S	S	S	S	S	S					**2.2**
		SRR619095				S	S	S	S	S	S	S	S	S	S	S								**0.8**
**Sidrauski *et al.*, 2015**	SRP053402	SRR1795425	n/a	n/a	n/a	S	S	S	S	S	S	S	S	S	S	S	S	S	n/a	n/a	n/a	n/a	n/a	**11.9**
		SRR1795426			S	S	S	S	S	S	S	S	S	S	S	S	S	S					n/a	**11.1**
		SRR1795427			S	S	S	S	S	S	S	S	S	S	S	S	S	S	S	S			n/a	**4.5**
		SRR1795428			S	S	S	S	S	S	S	S	S	S	S	S	S	S	S	S		n/a	n/a	**4.5**
		SRR1795429			S	S	S	S	S	S	S	S	S	S	S	S	S	S	S	S		n/a	n/a	**4.3**
		SRR1795430			S	S	S	S	S	S	S	S	S	S	S	S	S	S	S	S		n/a	n/a	**4.3**
		SRR1795431			S	S	S	S	S	S	S	S	S	S	S	S	S	S	S	S			n/a	**3.1**
		SRR1795432			S	S	S	S	S	S	S	S	S	S	S	S	S	S	S	S			n/a	**3.2**
		SRR1795433				S	S	S	S	S	S	S	S	S	S	S	S	S	S	S			n/a	**3.8**
		SRR1795434				S	S	S	S	S	S	S	S	S	S	S	S	S	S	S			n/a	**4.0**
		SRR1795435				S	S	S	S	S	S	S	S	S	S	S	S	S	S	S		n/a	n/a	**5.0**
		SRR1795436				S	S	S	S	S	S	S	S	S	S	S	S	S	S	S		n/a	n/a	**5.0**
		SRR1795437	S	S	S	S	S	S	S	S	S	S	S	S	S	S	S	S	S	S	S			**19.8**
		SRR1795438	S	S	S	S	S	S	S	S	S	S	S	S	S	S	S	S	S	S	S			**17.9**
		SRR1795439	S	S	S	S	S	S	S	S	S	S	S	S	S	S	S	S	S	S	S			**10.7**
		SRR1795440	S	S	S	S	S	S	S	S	S	S	S	S	S	S	S	S	S	S	S			**10.0**
**Stumpf *et al.*, 2013**	SRP029589	SRR970490	S	S	S	S	S	S	S	S	S	S	S	S	S	S	S	S	S	S	S			**0.8**
		SRR970538	S	S	S	S	S	S	S	S	S	S	S	S	S	S	S	S	S	S	S		n/a	**1.9**
		SRR970561	S	S	S	S	S	S	S	S	S	S	S	S	S	S	S	S	S	S	S			**5.4**
		SRR970565	S	S	S	S	S	S	S	S	S	S	S	S	S	S	S	S	S	S	S			**0.4**
		SRR970587	S	S	S	S	S	S	S	S	S	S	S	S	S	S	S	S				n/a	n/a	**4.0**
		SRR970588	S	S	S	S	S	S	S	S	S	S	S	S	S	S	S	S						**0.1**
**Subtelny *et al.*, 2014**	SRP033369	SRR1039861	n/a					S	S	S	S	S	S	S	S	S	S	S	n/a	n/a	n/a	n/a	S	**18.8**

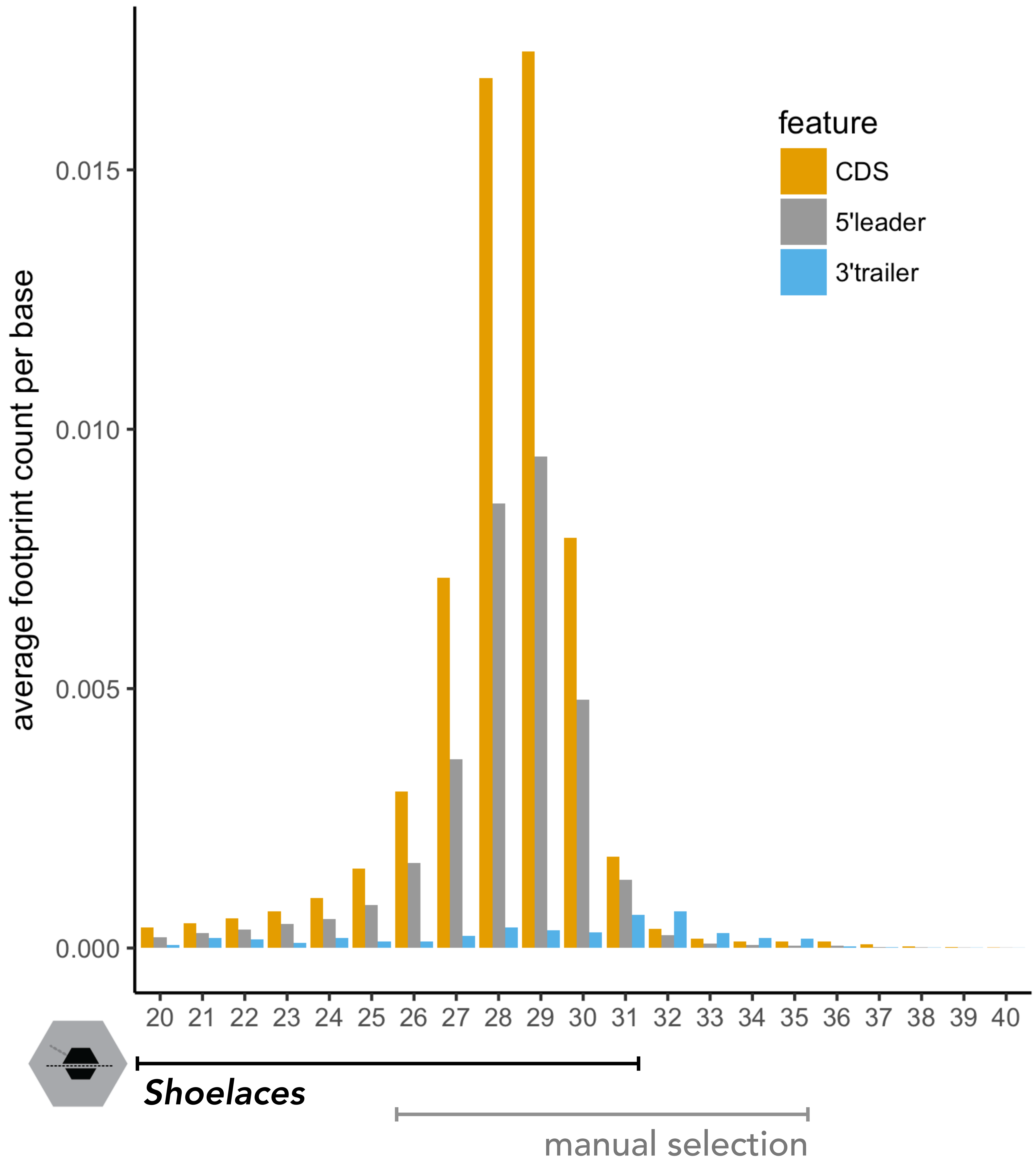

Additional figure 4
Average count of 5′ends of footprints per base in CDSs, 5′leaders and 3′trailers, stratified by fragment length for SRR493747 (Ingolia *et al.*, 2012). The fragments of lengths 20-31 exhibit periodicity (see Additional figure 1), and were therefore selected by Shoelaces, while in the original selection the reads of lengths 26-35 were kept. The selection performed by Shoelaces increases the count of translating footprints (CDSs and 5′leaders) and decreases the count of long footprints mapping to 3′trailers, which could be stemming from mRNA binding proteins, secondary structure or other sources of noise.

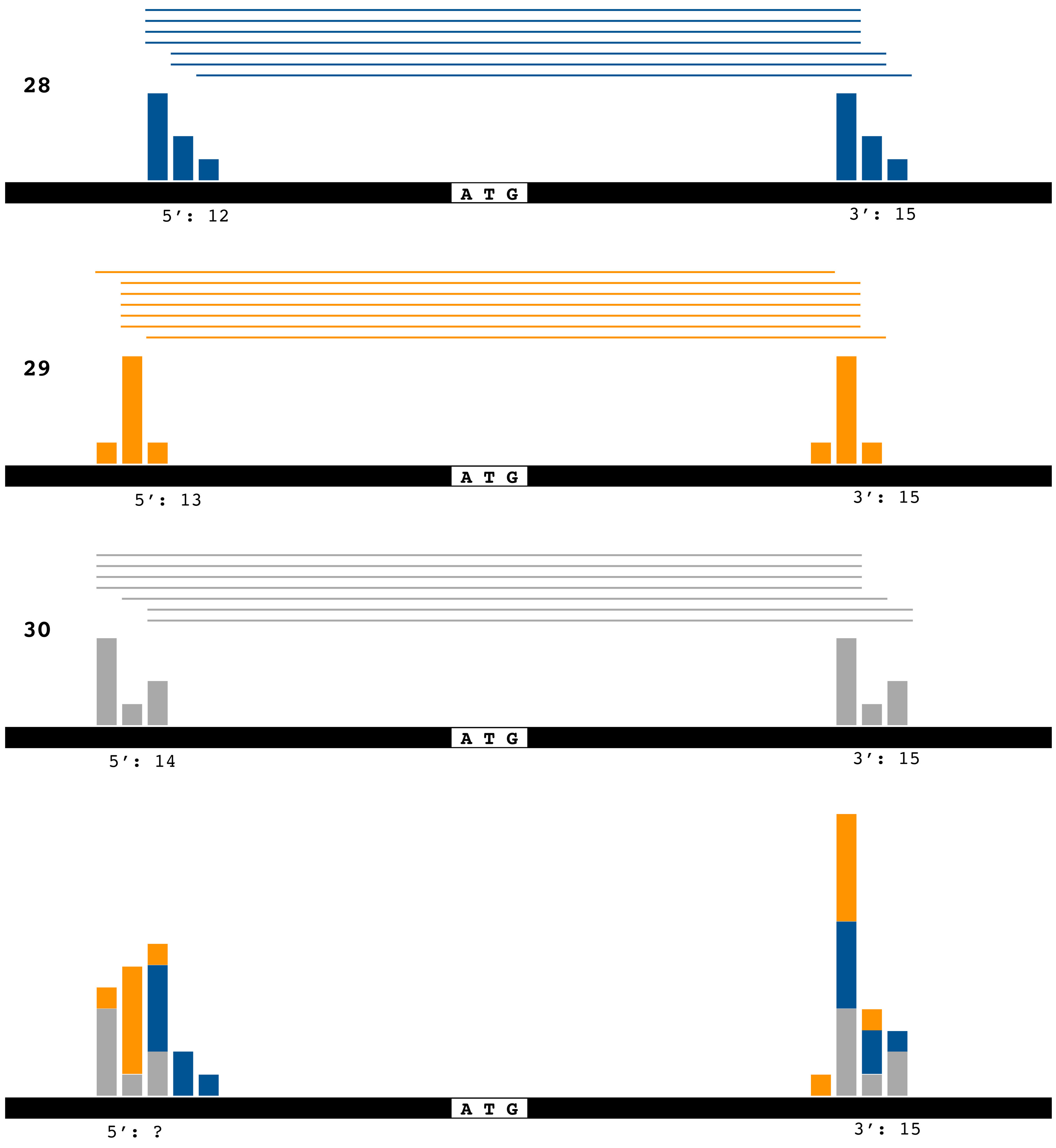

Additional figure 5

Calibrating offsets for each fragment length separately bypasses the need to consider both 5′ and 3′ assignment strategies, as both types of assignments are mathematically identical. Here, for the three footprint lengths of 28 (blue), 29 (orange) and 30 nucleotides (grey), the 5′ offsets to the first base of start codon are 12, 13 and 14 nt long respectively, while the 3′ offset is uniform and has length 15 nt. Have all the footprint lengths been aggregated (bottom panel), the profile of 5′ ends of footprints would have been ambiguous (Woolstenhulme *et al.*, 2015).
